# Genome-Wide Expression Profiling of Small RNAs in Indian Strain of *Rhizoctonia solani* AG1-1A Reveals Differential Regulation of milRNAs during Pathogenesis and Crosstalk of Gene Regulation

**DOI:** 10.3390/jof7070561

**Published:** 2021-07-14

**Authors:** Naresh Babu Prathi, Chagamreddy Venkata Durga Rani, Sena Munuswamy Balachandran, Vellaisamy Prakasam, Yeshala Chandra Mohan, Sanivarapu Nagalakshmi, Sunil K. Srivastava, Raman Meenakshi Sundaram, Satendra K. Mangrauthia

**Affiliations:** 1 Institute of Biotechnology, Professor Jayashankar Telangana State Agricultural University (PJTSAU), Rajendranagar, Hyderabad 500030, India; nareshseshu0962@gmail.com (N.B.P.); ranivenkata@yahoo.com (C.V.D.R.); drycmohan@gmail.com (Y.C.M.); 2 Indian Council of Agricultural Research (ICAR)-Indian Institute of Rice Research, Hyderabad 500030, India; balasena@yahoo.com (S.M.B.); vprakasam.iari@gmail.com (V.P.); nagalakshmi.s9@gmail.com (S.N.); r.sundaram@icar.gov.in (R.M.S.); 3 Department of Microbiology, Swami Shraddhanand College, University of Delhi, Alipur, Delhi 110036, India; sun27sri@gmail.com

**Keywords:** microRNA, sheath blight, *Oryzasativa*, fungi, strain, qRT-PCR

## Abstract

*Rhizoctonia solani* AG1-1A is a necrotrophic fungus that causes sheath blight disease in rice. The reliable resistant source against this phytopathogenic fungus is not available in the gene pool of rice. Better understanding of pathogen genomics and gene regulatory networks are critical to devise alternate strategies for developing resistance against this noxious pathogen. In this study, miRNA-like RNAs (milRNAs) of an Indian strain of *R. solani* were identified by deep sequencing of small RNAs. We identified 128 known and 22 novel milRNAs from 20,963,123 sequence reads. These milRNAs showed 1725 target genes in the fungal genome which include genes associated with growth, development, pathogenesis and virulence of *R. solani.* Notably, these fungal milRNAs showed their target genes in host (rice) genome also which were later verified by qRT-PCR. The host target genes are associated with auxin metabolism, hypersensitive response, defense genes, and genes related to growth and development of rice. Osa-vacuolar-sorting receptor precursor: Rhi-milR-13, Osa-KANADI1:Rhi-milR-124, Osa-isoflavone reductase: Rhi-milR-135, Osa-nuclear transcription factor Y:Rhi-milR-131, Osa-NB-ARC domain containing protein: Rhi-milR-18, and Osa-OsFBX438: Rhi-milR-142 are notable potential regulons of host target genes: fungal milRNAs that need to be investigated for better understanding of the crosstalk of RNAi pathways between *R. solani* and rice. The detailed expression analysis of 17 milRNAs by qRT-PCR was analysed during infection at different time points of inoculation, at different growth stages of the host, in four different genotypes of the host, and also in four different strains of fungi which revealed differential regulation of milRNAs associated with pathogenesis and virulence. This study highlights several important findings on fungal milRNAs which need to be further studied and characterized to decipher the gene expression and regulation of this economically important phytopathogen.

## 1. Introduction

*Rhizoctonia solani* AG1-1AKühn (Teleomorph: *Thanatephorus cucumeris* (A.B. Frank) Donk.) is a basidiomycete semi-saprotrophic soil-borne fungal pathogen that causes sheath blight disease in rice. In Eastern Asia, *R. solani* causes a loss of 6 million tons of rice grains per year [1]. India suffers 10% yield loss in rice due to sheath blight disease every year [2]. The pathogen is present in the form of sclerotia or mycelium, and severely affects high yielding, semi-dwarf, and nitrogen-responsive rice cultivars. Initial symptoms are noticed on leaf sheaths near water level. Warm and humid weather, dense planting, and high nitrogen inputs are the favourable conditions for its aggravation. Until now, rice germplasm immune to *R. solani* has not been known. Among several other factors, poor understanding of pathogen biology is one of the key factors that hamper the development of resistant cultivars either through breeding or transgenic approaches. Recently, efforts have been made to understand the regulation of the genome and transcriptome of *R. solani* during pathogenesis [3,4,5].

Clear understanding of pathogen genomics and gene regulatory networks is extremely important for drawing effective strategies for resistance development against diseases. One such example is the rice–*Xanthomonas oryzae* pv. *oryzae* (*Xoo*) pathosystem where deeper understanding of bacterial *TALE* (transcription activator-like effector) genes and their interaction with rice *SWEET* genes has helped developing durable and broad spectrum bacterial blight-resistant rice genotypes through marker-assisted breeding and genome editing [6,7,8,9]. Genomics has been extremely useful to facilitate host-induced gene silencing (HIGS) technology for the control of *Fusarium* diseases through targeted disruption of key genes [10]. Recently, Dong and Ronald (2019) [11] presented comprehensive information on pathogen genes used for disease resistance development through genetic engineering, RNAi, and genome editing. In addition to genetic resistance, fungal genomics is crucial for fungicide development by identifying potential fungicide targets, their validation and mode of action, as demonstrated in several pathosystems [12]. For more than a decade, small RNAs have taken central stage in the area of genomics due to their versatile functions in gene regulation and metabolic pathways [13,14].

Small RNAs (sRNAs) of <200 nucleotides are classified into different categories such as miRNA, siRNA, piwiRNA, snoRNA and t-RNAs etc. Recently, more sRNAs have been discovered based on their site of synthesis, binding proteins, and secondary structure and function, for example, trans-acting siRNAs (tasiRNAs) [15], repeat associated siRNAs (rasiRNAs) [16], heterochromatic small RNAs (hcRNAs) [17], and small scan RNAs (scnRNAs) [18] etc. MicroRNAs (miRNAs) are 21–24 nucleotide endogenous non-coding RNA molecules that work as defensive regulators in eukaryotes through post-transcriptional gene regulation. To date, numerous miRNAs have been discovered in plants and animals, but very few miRNAs have been reported in fungi [19]. Recent advancement in sequencing technologies and bioinformatics tools facilitated the identification of miRNAs in fungi [19,20,21]. MicroRNAs are critical regulators of host–pathogen interaction, and may help disease initiation and establishment [19,21,22,23].

In the model fungi *Neurospora crassa*, four different types of miRNA-like RNAs (milRNAs) processed through a dicer-independent pathway were identified. The proteins responsible for the production of these milRNAs are different from the plants and animals to a large extent [24]. Considering the essential functions of miRNAs in gene regulation and metabolic processes, there is a necessity to study the fungal genome encoded milRNAs which would give different insights and roles of microRNAs in pathogenesis and disease development. To date, very few milRNAs have been reported in plant pathogenic fungi, which could be due to the low accumulation of milRNAs or the small number of cells at certain infection stages, and also due to lesser attention of researchers. Recently, Meng et al. (2021) [25] identified virulence-associated milRNAs from *R. solani* AG1-IA infecting maize crop. With this perspective, our current study focussed on discovering the milRNAs in *R. solani* causing sheath blight disease in rice. Genome-wide small RNAs isolated from a pure culture of the Indian strain of *R. solani* AG1-1Awere sequenced to identify the known and novel milRNAs. Later, the targets of these milRNAs were predicted in *R. solani* and its host (rice) genome. Expression of 17 fungal milRNAs was analyzed in infected tissue of rice at different time points of inoculation, in different genotypes and growth stages of rice, and also in different strains of fungi. Expression analysis of target genes present in fungal and rice genome suggested that milRNAs are critical regulators of host–pathogen interaction.

## 2. Material and Methods

### 2.1. Fungal and Plant Material

In this study, four strains of *R. solani* AG1-IA were used. These strains were Wgl-2, Chn-1, Imph-2 and Lud-1 [26]. Four rice genotypes as a fungal host were used that include susceptible (TN1 and BPT5204) and resistant (Tetep and Pankaj) cultivars.

### 2.2. RNA Isolation and Sequencing

The highly virulent Indian strain of *R. solani* AG1-IA Wgl-2 was grown in growth medium having pectin as a carbon source. The fungal sclerotium was placed in liquid medium (0.7 g K_2_HPO_4_, 0.5 g KH_2_PO_4_, 0.5 g MgSO_4_, 0.01 g FeSO_4_, 0.001 g ZnSO_4_, and 10g pectin in 1 L distilled and autoclaved water) at 28 °C for 48–72 h as a still culture. The mycelium from three biological replicates was harvested and pooled to extract the RNA using TRIzol reagent (Invitrogen, Waltham, MA, USA). The quality of RNA was checked by Nanodrop (Thermo Fisher Scientific, Waltham, MA, USA), Bioanalyzer (Agilent 2100), and agarose gel electrophoresis to check the integrity, concentration, and contamination. After quality control (QC), the sequencing library was constructed by TruSeq Small RNA Library Preparation Kit (Illumina, CA, USA). The cDNA library was prepared by sequencing adaptor ligation, reverse transcription, PCR enrichment, purification and size selection. The QC of library was analyzed by Qubit 2.0 (preliminary library concentration), Agilent 2100 (insert size), and qPCR (effective concentration of library). The QC passed libraries were fed into HiSeq 2500 sequencer for sequencing.

### 2.3. milRNAs Identification in R. solani

The sequence data were analyzed to identify the milRNAs in the *R. solani* genome. Clean reads without adaptorcontamination were mapped to reference genome available in the RSIADB database [27]. Further non-coding sRNAs like rRNA, snRNA, snoRNA, and tRNAs were removed by using the Rfamdatabase (https://rfam.xfam.org/, accessed on 1 July 2017). The mRNA sequences or coding transcripts were filtered using RSIADB database. Later, miRNA prediction was undertaken using miREAP and miRDeep2 software for identification of both known and novel milRNAs. The secondary structures of these potential novel milRNA precursors were predicted by the RNAfoldWebServer program (http://rna.tbi.univie.ac.at/cgi-bin/RNAWebSuite/RNAfold.cgi, accessed on 1 July 2017). Putatively novel milRNAs were identified based on the following criteria: (1) mature milRNA sequence should be in either of the arm of hairpin structure, (2) number of mismatches 4 or less, (3) no loop or break in milRNA sequences, (4) asymmetric bulges should be minimal in size (one or two bases) and frequency (typically one or less), especially within the milRNA/milRNA* duplex [3,20,28].

### 2.4. The Target Gene Prediction in R. solani and Rice Genome

The target genes of milRNAs in the *R. solani* genome were predicted by using Miranda software [21] and the RSIADB database. The target genes of fungal encoded milRNAs in rice genome were predicted by using the psRNA Target database [29]. The default criteria of the target prediction tool were used for identification of milRNA target genes. 

### 2.5. Expression Analysis of Fungal milRNAs and Their Target Genes

Forty-five day old plants of rice cultivars TN1, Pankaj, BPT 5204, and Tetep were inoculated by *R. solani* AG1IA Wgl-2 strain. In parallel, TN1 was inoculated by three other strains, i.e., Chn-1, Imph-2 and Lud-1. For inoculation, the fungal sclerotium was placed in the sheath tissue of rice using a transparent cello tape. The sheath tissue was harvested from different rice genotypes (TN1, Pankaj, BPT 5204, and Tetep) infected by the Wgl-2 strain. Also, infected sheath tissue was harvested from TN1 at different time points of infection (18 h, 24 h, 48 h, 72 h, 96 h, and 5 days after inoculation) as well as from 45- (vegetative) and 80- (reproductive) day-old growth stages infected by the Wgl-2 strain. To analyze the strain specific milRNAs regulation, tissue was harvested from TN1 infected by three other strains (Chn-1, Imph-2, and Lud-1). Small RNAs and mRNAs were isolated from fungal infected rice tissue and pure culture of fungi (grown in potato dextrose agar medium) using the mirVana miRNA isolation kit (Ambion, TX, USA) according to the manufacturer’s instructions. cDNA synthesis of normalized sRNAs and mRNAs was performed using the miScript II RT kit (Qiagen, Hilden, Germany) and Improm-II reverse transcription system (Promega, Madison, WI, USA), respectively. The respective cDNA was used as template for milRNA and target gene quantification using miScript SYBR Green PCR kit (Qiagen, Germany) and SYBR Premix Ex-Taq (Takara, Shiga, Japan), respectively. Three biological replicates and two technical replicates were used for all the qRT-PCR experiments. Following the manufacturer instructions, all the kit reagents along with the milRNA and gene-specific primers (Table S1) were used for setting up the qRT-PCR reaction in a CFX96 Real-Time System (BIO-RAD, Hercules, CA, USA). The temperature profile was followed as described in previous report [30]. To ensure specificity of amplified product, melt curve analysis and agarose gel electrophoresis were used. To quantify the relative expression of milRNAs and target genes, the comparative threshold cycle (C_T_) method was followed. The relative expression was calculated from 2^−ΔΔCt^. The standard error was calculated as reported previously [31].

## 3. Results

### 3.1. Sequence Statistics

The milRNAs encoded by the *R. solani* AG1 IA genome were identified through Illumina deep sequencing of small RNAs. All the sequence reads were aligned to the reference genome of *R. solani* available in the RSIADB database [27] and the mapped clean reads were further used to identify the known and novel milRNAs. After deep sequencing of *R. solani* small RNAs 20,963,123 raw reads and 18,084,903 clean reads were obtained. The clean reads were used to filter 1,231,411 reads of 18–24 nts size of which 185,760 were unique reads. 2574 reads of *R. solani* aligned with hairpin sequences of known milRNAs [21], while 619,582 reads were utilized for prediction of novel milRNAs. 

### 3.2. Identification of Known and Novel milRNAs

**Figure 1:**
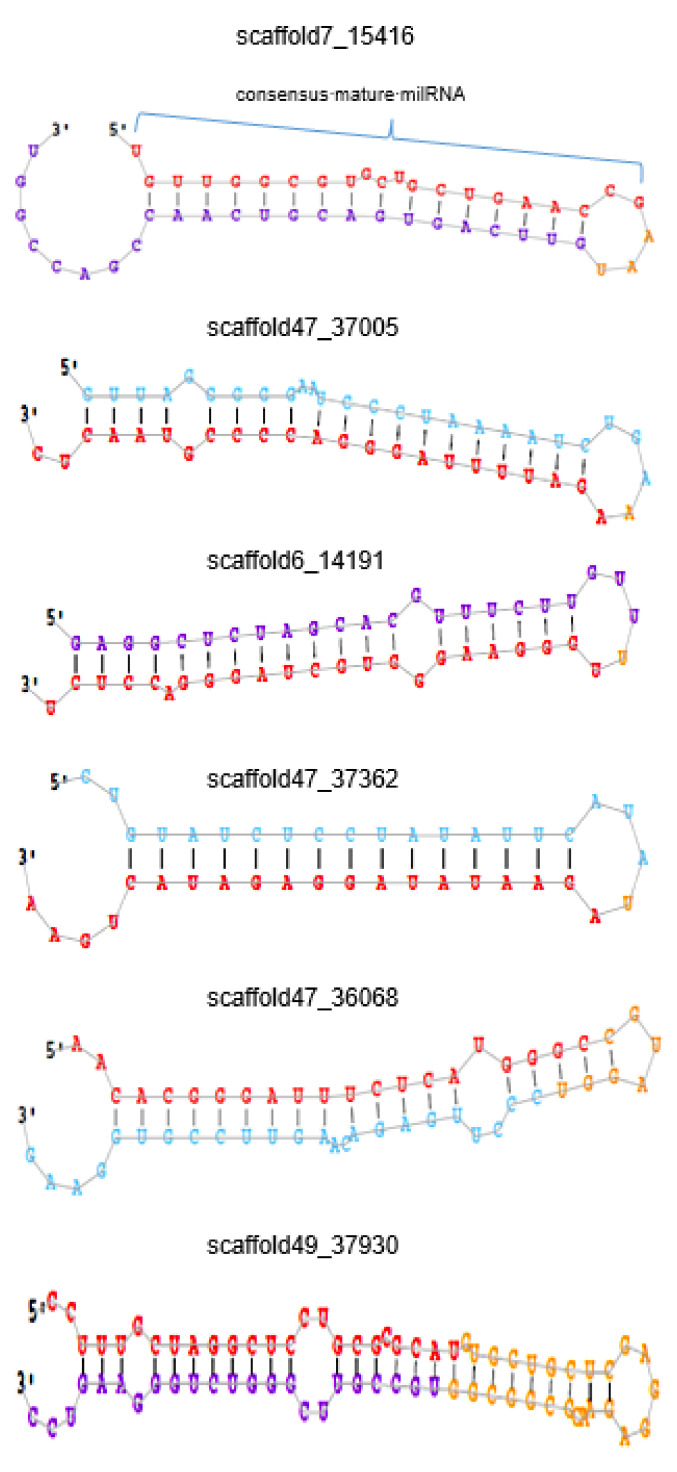
Depiction of representative novel milRNAs identified in this study. Red colour nucleotides indicate the sequence of mature milRNAs. Blue, purple, and golden colours are the backbone of novel milRNAs.

128 known milRNAs of *R. solani* were identified in Indian strain of the pathogen (Table S2). Among these, Rhi-milR-16, Rhi-milR-22, Rhi-milR-35, Rhi-milR-57, Rhi-milR-91, and Rhi-milR-112 showed higher read count than the others. Twenty two novel milRNAs were predicted among which scaffold13_22000, scaffold47_37005, scaffold47_37523, scaffold47_37559, scaffold47_37341, scaffold47_37407, and scaffold47_36418 showed higher read account than others. Notably, scaffold13_22000 showed >6000 read count (Table 1). The secondary structure of a few representative novel milRNAs is shown in Figure 1.

### 3.3. Identification of Target Genes in R. solani Genome

We identified 1725 target genes of 128 known and 22 novel milRNAs in the *R. solani* genome (Table S3). The known milRNAs targeting the genes encoding carbohydrate active enzymes, secretory proteins, and other critical proteins associated with fungal biology are listed (Table 2 and Table S4). Also, the target genes of novel milRNAs were predicted (Table 3). Notably, Scaffold47_36931 showed the highest number (12) of target genes. Rhi-milR-141, Rhi-milR-120, Rhi-milR-41, and Rhi-milR-91 showed pectin esterase and pectate lyase domain-containing protein genes as their targets. 

### 3.4. Identification of R. solani-milRNAs Target Genes in Oryza Sativa Genome

In addition to identifying the gene targets of fungal encoded milRNAs in fungal genome, target genes of these milRNAs were also searched in its host genome. Interestingly, 22 known and 2 novel milRNAs showed their target genes in the rice genome. Some of these notable target genes:milRNA regulatory pairs are Osa-KANADI1:Rhi-milR-124, Osa-vacuolar-sorting receptor precursor:Rhi-milR-13, Osa-nuclear transcription factor Y:Rhi-milR-131, Osa-isoflavone reductase: Rhi-milR-135, Osa-OsFBX438: Rhi-milR-142, Osa-NB-ARC domain containing protein: Rhi-milR-18, Osa-plastocyanin-like domain containing protein:Rhi-milR-22, Osa-cytochrome P450 81E1: Rhi-milR-26, Osa-response regulator receiver domain containing protein: Rhi-milR-43, Osa-serine/threonine-protein kinase receptor precursor: Rhi-milR-51, Osa-OsMADS58: Rhi-milR-66, and Osa-extra-large G-protein-related: Rhi-milR-81. Among the novel miRNAs, Scaffold130_44481 milRNA targets the Osa-plastocyanin-like domain containing protein while scaffold47_37559 targets transferase family protein (Table 4). 

### 3.5. qRT-PCR Expression Analysis of milRNAs

Seventeen milRNAs (known and novel) were selected based on read count, target genes in *R. solani*, and also based on the target genes in rice. The relative expression of fungal encoded milRNAs during host (rice) infection was analyzed at different time points of infection, different age and genetic makeup of host tissue, and also in different strains of fungi. The number of expressed milRNAs varied in different experiments, therefore, only those milRNA were included which showed expression in at least one sample. 

#### 3.5.1. At Different Time Points of Inoculation

**Figure 2:**
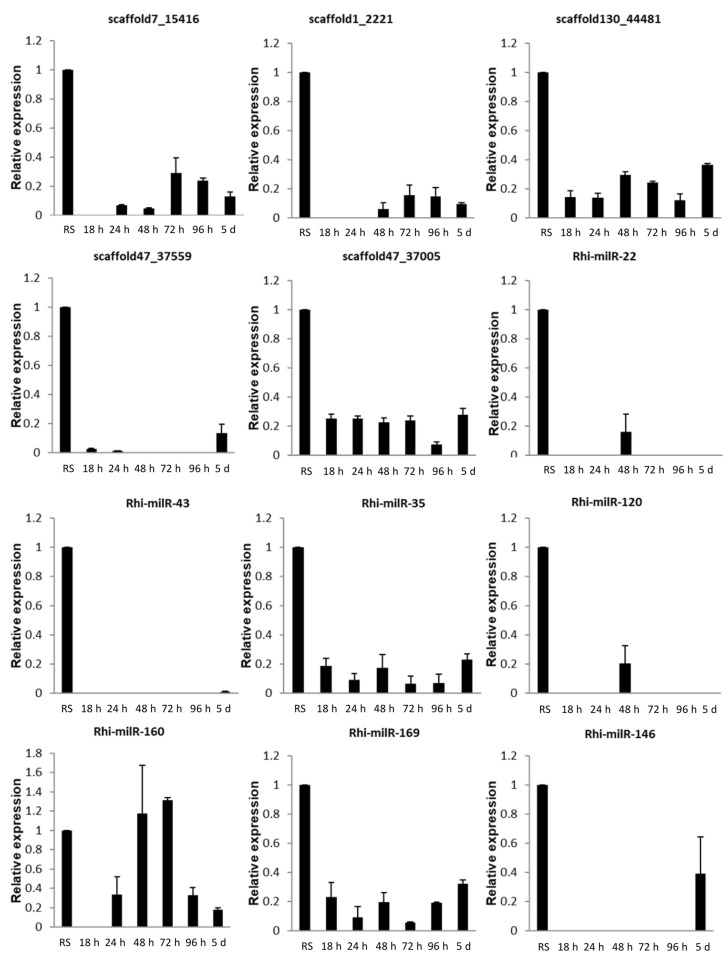
qRT-PCR expression analysis of milRNAs at different time points after fungal inoculation. X-axis: samples (RS: *R. solani* cultured in potato dextrose agar medium, 18 h to 5 d: *R. solani* inoculated sheath tissue of TN1 harvested at different time points); Y-axis: Expression level of the individual milRNA relative to its expression in *R. solani* grown in PDA. Error bars indicate the mean ± S.E. of three biological replicates.

TN1, the susceptible rice cultivar, was inoculated with *R. solani* to study the expression levels of fungal-encoded milRNAs at different time points of infection. While comparing with the expression of milRNAs in *R. solani* grown in PDA medium, all the milRNAs except Rhi-milR-160 showed down-regulation during the rice infection at all time points. Furthermore, the milRNAs were differentially regulated at different time point of infection. Novel milRNAs scaffold130_44481 and scaffold47_37005, and known milRNAs Rhi-milR-35 and Rhi-milR-169 expressed at all time points, while scaffold7_15416 and Rhi-milR-160 expressed at all the time points except at 18 h. Among all the milRNAs analyzed, only Rhi-milR-160 showed up-regulation (at 48 h and 72 h time points). Scaffold1_2221 expressed at 48 h, 72 h, 96 h and 5d while scaffold47_37559 showed expression at 18 h, 24 h, and 5 days. Expression of Rhi-milR-22, Rhi-milR-43, Rhi-milR-120 and Rhi-milR-146 was observed exclusively at a specific time point of infection, i.e.,72 h, 5 days, 48 h and 5 days respectively. Notably, the Rhi-milR-43 showed down-regulation at 5 days. While analyzing the expression of milRNAs at different time points, most of the milRNAs showed expression at 5 days (Figure 2).

#### 3.5.2. In Different Growth Stages of Host Plant

**Figure 3:**
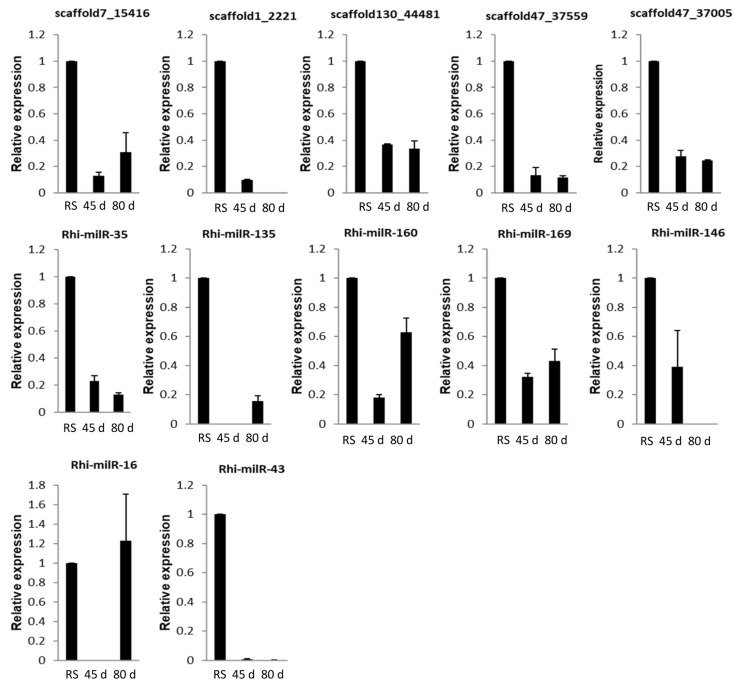
qRT-PCR expression analysis of milRNAs in sheath tissue of 45 days and 80 days old TN1 plants after *R. solani* inoculation. X-axis: samples; Y-axis: Expression level of the individual milRNA relative to its expression in *R. solani* grown in PDA. Error bars indicate the mean ± standard error (SE) of three biological replicates.

The expression of fungal encoded milRNAs was influenced by the growth stage of its host. All the milRNAs except Rhi-milR-16 showed down-regulation at both the growth stages during infection when compared with the expression level of milRNAs in fungi grown in PDA medium. Among the two growth stages, scaffold7_15416, Rhi-milR-160, and Rhi-milR-169 showed more expression at reproductive stage (80 days old) of host than vegetative (45 days old). Rhi-milR-43 showed down-regulation in a 45-day-old host and the expression levels further declined in an 80-day-old host. Interestingly, Rhi-milR-16 did not show expression in a 45-day-old host but was up-regulated in an 80-day-old host; whereas Rhi-milR-146 did not show expression in 80 days old host but was down-regulated in a 45-day-old host. Scaffold1_2221 and Rhi-milR-135 also showed stage specific expression, i.e., down-regulation in 45 and 80-day-old hosts, respectively. Scaffold7_15416, scaffold130_44481, scaffold47_37559, scaffold47_37005, Rhi-milR-43, Rhi-milR-35, Rhi-milR-160, and Rhi-milR-169 showed down-regulation in both 45-dayold and 80-day-old hosts (Figure 3).

#### 3.5.3. In Different Rice Genotypes

**Figure 4:**
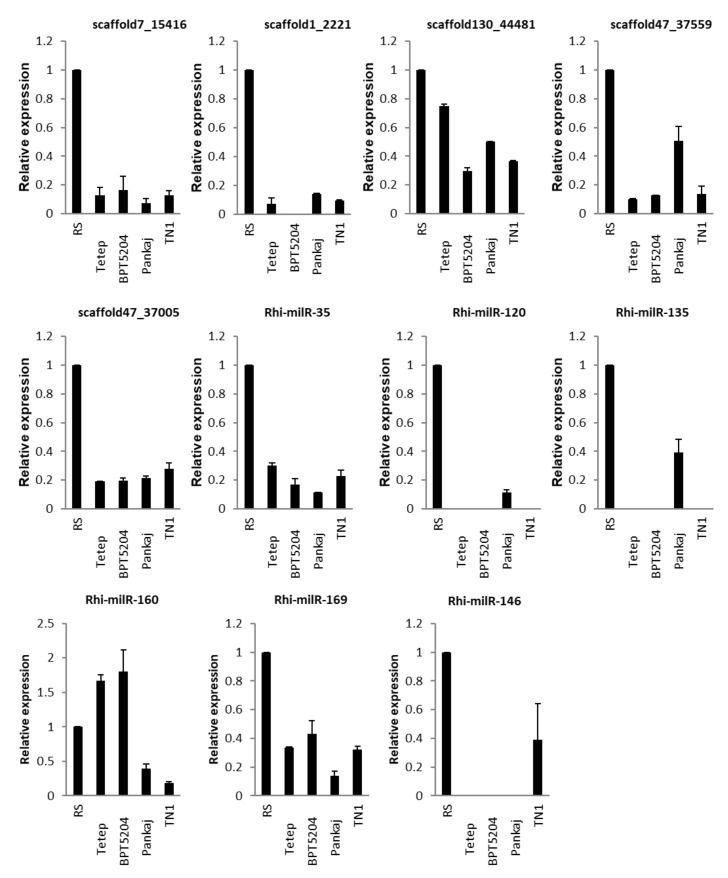
qRT-PCR expression analysis of milRNAs in different rice genotypes after *R. solani* inoculation. X-axis: samples (*R. solani* inoculated sheath tissue of four rice genotypes); Y-axis: Expression level of the individual milRNA relative to its expression in *R. solani* grown in PDA. Error bars indicate the mean ± S.E. of three biological replicates.

Expression of fungal-encoded milRNAs was analysed during the infection of four different genotypes of rice, i.e., Tetep, BPT5204, Pankaj, and TN1. Forty-five day-old plants of these genotypes were inoculated with Wgl-2 strain of *R. solani* and samples were collected after 5 days of inoculation. All the milRNAs except Rhi-milR-160 showed down-regulation during infection of four rice genotypes when compared with the expression level of milRNAs in fungi grown in PDA medium. Seven milRNAs: scaffold7_15416, scaffold130_44481, scaffold47_37559, scaffold47_37005, Rhi-milR-35, Rhi-milR-160, and Rhi-milR-169 showed expression in all the genotypes. These milRNAs were down-regulated during infection of rice genotypes. Among these, expression of scaffold130_44481 was more in tolerant (Tetep and Pankaj) than the susceptible (TN1 and BPT5204) rice genotypes. Expression of scaffold47_37559 was greater in Pankaj than the other three genotypes while, Rhi-milR-169 expression was lesser in Pankaj than the other three genotypes. Rhi-milR-160 showed up-regulation in Tetep and BPT5204 but down-regulation in Pankaj and TN1. Rhi-milR-120 and Rhi-milR-135 showed exclusive expression in Pankaj while Rhi-milR-146 showed exclusive expression in TN1. Scaffold1_2221 expressed in all genotypes except BPT5204 (Figure 4). 

#### 3.5.4. In Different Strains of Fungus

**Figure 5:**
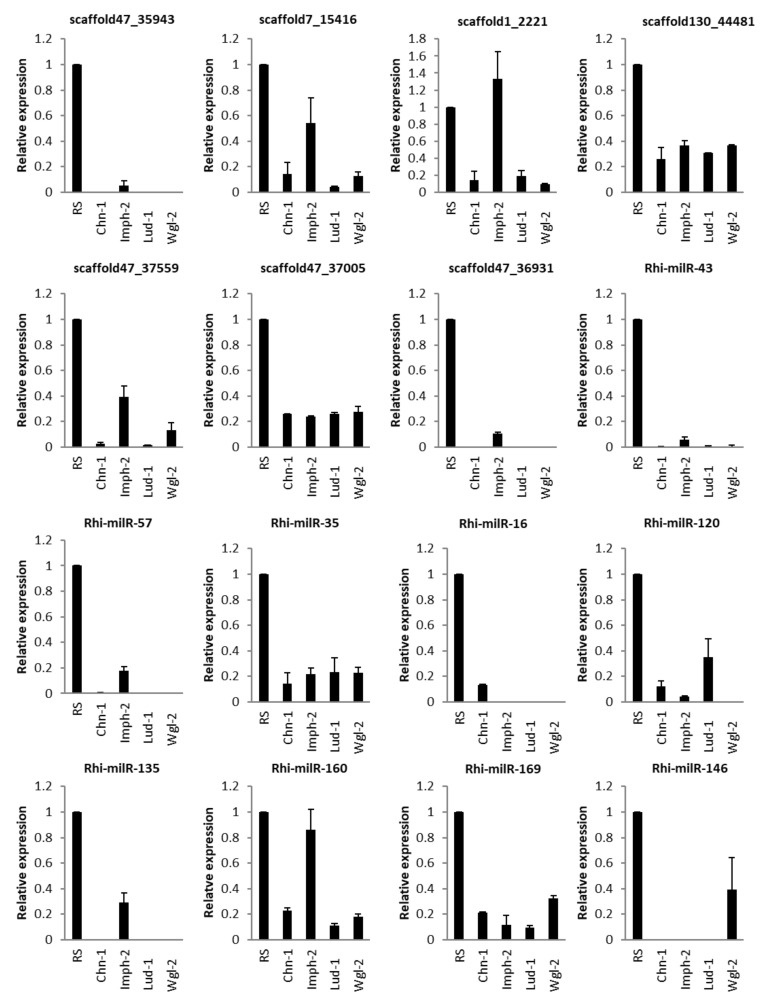
qRT-PCR expression analysis of milRNAs in sheath tissue of TN1 after inoculation with different strains of *R. solani*. X-axis: samples (sheath tissue of TN1 inoculated with four different strains of *R. solani*); Y-axis: Expression level of the individual milRNA relative to its expression in *R. solani* grown in PDA. Error bars indicate the mean ± S.E.

Expression of milRNAs of four different fungal strains was analysed during the infection of the TN1 rice genotype. All the milRNAs except scaffold1_2221 showed down-regulation during infection in all fungal strains when compared with the expression level of respective milRNAs in fungi grown in PDA medium. Novel milRNAs scaffold7_15416, scaffold1_2221, scaffold130_44481, scaffold47_3559, scaffold47_37005 and known milRNAs Rhi-milR-35, Rhi-milR-160 and Rhi-milR-169 showed expression in all the four strains of fungi while infecting its host. Among these, scaffold7_15416, scaffold1_2221, scaffold47_37559, and Rhi-milR-160 showed more expression in Imph-2 strain than three other fungal strains. Furthermore, scaffold47_35943, scaffold47_36931, Rhi-milR-43, Rhi-milR-57, and Rhi-milR-135 showed expression only in Imph-2 strain while infecting TN1. Notably, scaffold1_2221 was up-regulated in Imph-2 strain but down-regulated in the other three strains. Rh-milR-16 and Rhi-milR-146 showed exclusive expression in Chn-1 and Wgl-2 strains, respectively. Rhi-milR-120 did not show expression in the Wgl-2 strain (Figure 5).

#### 3.5.5. Expression Analysis of Fungal and Plant Target Genes 

**Figure 6:**
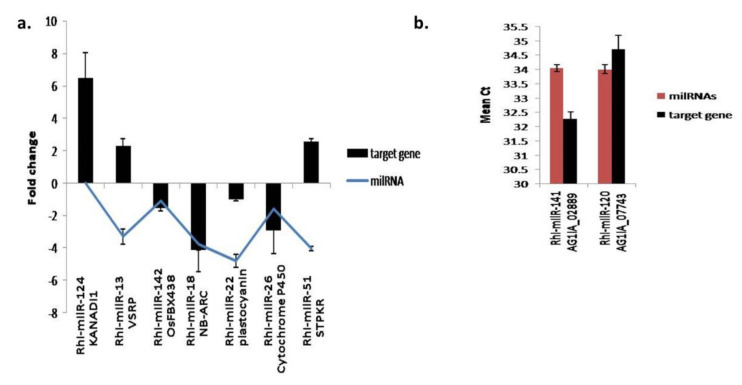
Expression analysis of target genes (**a**) qRT-PCR expression analysis of fungal milRNAs and their respective plant target genes. (**b**) qRT-PCR expression analysis of fungal milRNAs and their respective fungal target genes.

Expression of seven plant target genes and two fungal target genes was analysed in *R. solani*-infected rice (TN1) tissue. Along with the target genes, the expression of the respective regulatory milRNA was also analysed. Among the plant target genes, the regulons Osa-KANADI1:Rhi-milR-124, Osa-vacuolar-sorting receptor precursor:Rhi-milR-13, Osa-plastocyanin-like domain containing protein:Rhi-milR-22, and Osa-serine/threonine-protein kinase receptor precursor: Rhi-milR-51 showed anticipated reciprocal trend of expression. In these cases, the fungal encoded milRNAs and their respective plant target genes showed opposite trend of expression during the disease development (Figure 6a). Similarly, fungal genes AGA1IA_02889 and AGA1IA_07743 and their regulatory milRNAs Rhi-milR-141 and Rhi-milR-120 showed a contrasting expression pattern in infected tissue of rice (Figure 6b).

## 4. Discussion

MicroRNAs are regulatory genes that play critical roles in cellular processes of almost all eukaryotes [14]. It was believed that fungi do not possess microRNAs until the discovery of miRNA-like small RNAs (milRNAs) produced through at least four different biogenesis pathways in model fungi *N. crassa* [6,32]. Compared to plants and animals, there are very limited reports on milRNAs of phytopathogenic fungi which are primary agents causing diseases and huge loss to agriculture crops. In the absence of resistance in the rice germplasm against *R. solani*, it is extremely important to decipher the gene regulatory networks and biology of pathogens to devise effective strategies for controlling the menace of sheath blight disease in rice [31,33,34,35]. We analyzed the milRNAs of sheath blight disease causing pathogen *R. solani* AG1 IA by RNAseq of small RNAs. A total of 128 known and 22 novel milRNAs were identified. Most of the milRNAs showed low abundance which is accordance with earlier study of fungal milRNAs [36]. Notably, known milRNAs-Rhi-milR-16, Rhi-milR-22, Rhi-milR-35, Rhi-milR-57, Rhi-milR-91, and Rhi-milR-112 and novel milRNAs-scaffold13_22000, scaffold47_37005, scaffold47_37523, scaffold47_37559, scaffold47_37341, scaffold47_37407, and scaffold47_36418 showed more abundance than other microRNAs suggesting their significant role in fungal gene regulation. The novel milRNA scaffold13_22000 showed >6000 read count which emphasizes that several novel players identified in this study might regulate fungal genome. Earlier studies suggested that such novel milRNAs may have crucial roles in pathogenesis and virulence of *R. solani* [21,25]. 1725 target genes of 128 known and 22 novel milRNAs were identified that are involved in various metabolic and developmental processes of fungi indicating that milRNAs are major regulators of the *R. solani* genome. The role of milRNAs in fungal growth and development has been recently described in *Sclerotinia sclerotiorum* [37]. A number of milRNAs were identified in the soil-borne fungal pathogen *Verticillium dahlia* causing wilt diseases in several agriculture crops. One of the milRNAs (VdmilRNA1) was demonstrated to mediate epigenetic repression of a virulence gene (VdHy1) in pathogenic fungi [38]. Furthermore, the role of milRNAs (CmmilR4 and CmilR16) in fruiting body development was demonstrated in *Cordyceps militaris* [39]. The milRNAs-Rhi-milR-141, Rhi-milR-120, Rhi-milR-41, and Rhi-milR-91 target pectinesterase and pectate lyase domain-containing protein genes signifying their crucial role in plant cell wall degradation and pathogenesis. milRNAs of *R. solani* targeting carbohydrate active enzymes and other fungal genes including those involved in virulence and pathogen–host interaction were reported recently [25]. The genes encoding plant cell wall degrading enzymes of *R. solani* play significant role in disease initiation and establishment [4]. *Vm*-milR16 of *Valsamali* regulates the expression of pectinase genes during *V. Mali*–host interaction [40]. 

Interestingly, *R. solani* milRNAs showed their targets in the host (rice) genome also. A similar observation was made on *R. solani* targeting maize genes [25]. Recently, cross kingdom regulation of genes by miRNAs during host–pathogen interaction has been demonstrated in several pathosystems [41]. Bidirectional cross-kingdom transport of microRNAs or small RNAs through naked form, combined with RNA-binding proteins or enclosed by vesicles between fungal pathogens and its host plants has been reported [42]. In an interesting observation, Cui et al. (2019) [43] demonstrated that bba-milR1 encoded by pathogenic fungus *Beauveria bassiana* hijacks the RNA-interference machinery of a host (mosquito) to attenuate host immunity and facilitate infection. The fungus *Sclerotinia sclerotiorum* was reported to produce at least 374 distinct highly abundant sRNAs during infection of *Arabidopsis thaliana* and *Phaseolus vulgaris*. Targets of these small RNAs were significantly down-regulated during infection in *A. thaliana* [44].

Rhi-milR-124 targets Osa-KANADI1. In *Arabidopsis*, KANADI1 acts as a transcriptional repressor of genes involved in auxin biosynthesis, auxin transport, and auxin response [45]. Fungal pathogens control the auxin levels and auxin signalling pathways that significantly influence the defense network in plants [46]. In the wheat–*Zymoseptoria tritici* pathosystem, Ma et al. [47] showed that as an immune response, the fungal infection induced wheat small RNAs that regulate auxin-related genes. Rhi-milR-13 regulates Osa-vacuolar-sorting receptor (VSR) of rice which is responsible for targeting defense-related soluble proteins to the vacuole. Plant vacuoles play an important role in defense by releasing hydrolytic enzymes and antimicrobial compounds upon pathogen infection that leads to programmed cell death due to hypersensitive response [48]. Suppression of rice VSR by *R. solani* milRNA might suppress the plant defense by inhibiting the hypersensitive response. Notably, the target probability in the case of Osa-vacuolar-sorting receptor precursorRhi-milR-13 is very high with expectedvalue of 1.0. Therefore, further probing of this milRNA-target gene regulation network can add significant information on host-pathogen molecular cross-talk. Rhi-milR-131 targets nuclear transcription factor Y (NFY) subunit of rice which is also regulated by the host miRNA, i.e., Osa-miR169. The over-expression of Osa-miR169 in rice led to repression of NFY resulting in hyper-susceptibility of transgenic rice against blast fungus *Magnaportheoryzae* [49]. Repression of NFY by Rhi-milR-131 may weaken the defense response of rice against *R. solani*. Other host target genes of fungal milRNAs included isoflavone reductase, OsFBX438-F-box domain containing protein, NB-ARC domain containing protein, serine/threonine-protein kinase receptor, and rabGAP/TBC domain-containing protein which are involved in plant defense against pathogens [50,51,52,53,54]. *R. solani*-encoded milRNAs target maize defensegenesduring infection and negatively regulate resistance of maize [25]. Notably, the regulons such as Osa-KANADI1:Rhi-milR-124, Osa-vacuolar-sorting receptor precursor:Rhi-milR-13, Osa-plastocyanin-like domain containing protein:Rhi-milR-22, and Osa-serine/threonine-protein kinase receptor precursor: Rhi-milR-51 were verified by analysing their expression through qRT-PCR during host–fungal interaction and disease development. Recent reports demonstrate a role of cross-kingdom RNAi in regulation of genes associated with pathogen virulence and host resistance. For example, small RNAs of phytopathogenic fungi *Botrytis cinerea* trigger the silencing of immunity-related genes of its hosts *A. Thaliana* and *Solanum lycopersicum* [55]. A similar observation has also recently been reported inthe case of *Puccinia triticina* and *P. striiformis* [56,57]. In addition to plant defense genes, fungal milRNAs showed their target as genes associated with growth and the development of the host. 

This study provides a first comprehensive analysis of milRNAs expression during pathogen infection as the expression of 17 milRNAs was analysed at different time points of infection, at different growth stages of the host, in different genotypes (with different genetic make-up) of host, and also in different strains of fungi. The down-regulation of all milRNAs except Rhi-milR-160 was noticed at all the time points of infection suggesting the induction of their target genes during the disease initiation and establishment. The differential regulation of milRNAs at different time points of infection suggests that fungal genes are regulated by milRNAs during disease initiation and progression. Scaffold130_44481, scaffold47_37005, Rhi-milR-35, and Rhi-milR-169 showed expression throughout all time points of infection indicating their central role in infection. Expression of Rhi-milR-22, Rhi-milR-43, Rhi-milR-120, and Rhi-milR-146 was observed specifically at 72 h, 5d, 48 h, and 5d respectively, suggesting that fungi recruit specific milRNAs as the infection progresses. During the infection and disease progression, *R. solani* requires mycotoxin production and penetration of mycelium inside the host cells. Studies in *Aspergillus flavus* and *Fusarium oxysporum* f. sp. *Niveum* highlighted the significant roles of milRNAs in the mycelium growth and mycotoxin biosynthesis [22,58]. Lin et al. (2016) [21] analysed the differential expression pattern of *R. solani* milRNAs during infection and suggested their roles in disease initiation and establishment [58]. Differential regulation of milRNAs during sclerotial development in *Sclerotinia sclerotiorum* was observed by analyzing small RNAs at different time points [59].

At the time of infection, expression of scaffold7_15416, Rhi-milR-160, and Rhi-milR-169 was more at the reproductive stage than vegetative stage of rice while Rhi-milR-16 did not show expression in vegetative stage but was up-regulated in the reproductive stage. By contrast, Rhi-milR-146 was down-regulated in the vegetative stage but no expression was observed in the reproductive stage. These results suggest that growth stage of host plant influences the milRNAs expression of fungi. Scaffold1_2221 was down-regulated in the vegetative stage while Rhi-milR-135 was down-regulated in reproductive stage, indicating their specific role during pathogenesis of different-aged hosts. Scaffold7_15416, scaffold130_44481, scaffold47_37559, scaffold47_37005, Rhi-milR-43, Rhi-milR-35, Rhi-milR-160, and Rhi-milR-169 were down-regulated at both the growth stages signifying their essential roles during infection.

Expression of seven-milRNAs-scaffold7_15416, scaffold130_44481, scaffold47_37559, scaffold47_37005, Rhi-milR-35, Rhi-milR-160, and Rhi-milR-169 was down-regulated while infecting the four rice genotypes suggesting their key roles in regulating the genes during infection. Expression of scaffold130_44481 and scaffold47_37559 was greater in tolerant genotypes than susceptible rice genotypes while Rhi-milR-120 and Rhi-milR-135 showed exclusive expression in Pankaj indicating that regulation by milRNAs is significantly influenced by host genetic architecture. Expression of other milRNAs also varied in different genotypes of its host.

Expression of scaffold7_15416, scaffold1_2221, scaffold130_44481, scaffold47_3559, scaffold47_37005, Rhi-milR-35, Rhi-milR-160, and Rhi-milR-169 was observed in all the four strains of fungi while infecting its host suggesting their primary role in infection of the host. Notably, expression of scaffold7_15416, scaffold1_2221, scaffold47_37559, and Rhi-milR-160 was greater in Imph-2 strain while scaffold47_35943, scaffold47_36931, Rhi-milR-43, Rhi-milR-57, and Rhi-milR-135 were exclusively expressed in Imph-2 strain indicating that different strains of a fungus have evolved differential milRNA mediated gene regulation. Imph-2 and Chn-1 are mild strains of *R. solani* [26]. Rh-milR-16 showed exclusive expression in Chn-1 while Rhi-milR-146 showed exclusive expression in the Wgl-2 strain. Expression pattern of milRNAs in this study indicate that milRNAs may play critical role in determining the virulence of fungal strains. *Vm*-milR16 of the phytopathogenic fungus *V. mali* regulates the expression of virulence genes [40].

Fungal genome-encoded microRNA-like RNAs are relatively less explored genetic elements that play a major role in regulation and expression of genes associated with growth, development, pathogenesis, and virulence. We made a comprehensive effort by combining RNAseq and qRT-PCR assays to decipher the milRNAs of an Indian strain of *R. solani* AG1-1A causing sheath blight disease of rice. Several milRNA candidates targeting key genes of *R. solani* were identified which need to be further characterized to understand their specific biological roles. The novel milRNAs are significant additions to understanding of the complexity of gene regulation of *R. solani.* The novel milRNA scaffold13_22000 showing very high expression needs to be characterized for its role in pathogen–host interaction and fungal metabolism. Further probing the regulation of rice genes by fungal milRNAs will be a milestone in understanding the molecular basis of host–pathogen interaction. Some of the gene regulatory networks such as Osa-KANADI1:Rhi-milR-124, Osa-vacuolar-sorting receptor precursor:Rhi-milR-13, and Osa-nuclear transcription factor Y:Rhi-milR-131 can be studied on priority. Also, the better understanding regulation of rice defense genes by *R. solani* milRNAs may help in executing more effective strategies against this deadly plant pathogen. Detailed expression analysis of milRNAs at different time points of infection, at different growth stages of hosts, in different genotypes of hosts, and in different strains of fungi provide ample information to plan future studies for understanding the gene regulation mechanism of *R. solani*. Our results indicate that milRNAs may contribute to almost all the metabolic processes of the *R. solani* pathogen.

## Figures and Tables

**Table 1:** Novel milRNAs predicted from small RNA sequence data of *Rhizoctonia solani* AG1-1A Wgl-2 strain.

S. No.	Novel milRNA	miRDeep2 Score	Estimated Probability That the miRNA Candidate Is a True Positive	Total Read Count	Consensus Mature Sequence	Precursor Coordinate
1	scaffold47_37407	193.1	70 +/− 24%	379	aaucccuaggaucuccacuug	scaffold47:62019..62062:−
2	scaffold47_37523	193	70 +/− 24%	379	aaucccuaggaucuccacuug	scaffold47:127383..127426:−
3	scaffold47_37341	192.9	70 +/− 24%	379	aaucccuaggaucuccacuug	scaffold47:26292..26335:−
4	scaffold47_36931	30.3	70 +/− 24%	80	Uggacaauguucugugggguu	scaffold47:118388..118435:+
5	scaffold145_44745	17.3	70 +/− 24%	37	Gacuaaucagcguugggcgcauuu	scaffold145:16023..16102:+
6	scaffold6_14191	6	66 +/− 23%	11	Ugggaagggugcuagggaccucu	scaffold6:504695..504742:−
7	scaffold10_19054	1.3	35 +/− 26%	3	Ugccgacugugcucccgcccaucg	scaffold10:751369..751434:−
8	scaffold1_2221	0.9	16 +/− 15%	5	Cagcacacuggaccgagagcucu	scaffold1:3047379..3047427:+
9	scaffold47_37005	0.8	16 +/− 15%	249	Agauuuuagggaccccguaacuc	scaffold47:124971..125020:+
10	scaffold12_20737	0.8	16 +/− 15%	27	gaguuuuggacuggcaccc	scaffold12:518031..518072:+
11	scaffold7_15416	0.6	16 +/− 15%	2	uguuggcgugcugcugaaccg	scaffold7:443987..444035:−
12	scaffold47_35943	0.4	16 +/− 15%	29	Aaggaaauucucguagggcuucu	scaffold47:16996..17045:+
13	scaffold47_36418	0.4	16 +/− 15%	94	Uuucuauggaccaccacgaguacu	scaffold47:65345..65392:+
14	scaffold49_37930	0.4	16 +/− 15%	2	Ccuuugcuaggcuccugcgcgcau	scaffold49:58540..58612:+
15	scaffold48_37687	0.3	16 +/− 15%	14	Ucucgaagcgcgacucuguccuu	scaffold48:142724..142771:+
16	scaffold30_30807	0.3	16 +/− 15%	30	Agcgcaacucgaccucugaucacg	scaffold30:89871..89933:−
17	scaffold34_32450	0.2	16 +/− 15%	52	Aucgcugacugcgguguccucu	scaffold34:218833..218913:−
18	scaffold13_22000	0	16 +/− 15%	6028	Aaggugccggaauauacgcucau	scaffold13:463575..463652:−
19	scaffold130_44481	0	16 +/− 15%	2	ccgugcaacggacgaucgac	scaffold130:10887..10931:−
20	scaffold47_37559	0	16 +/− 15%	148	Cguggacgggccgcauccc	scaffold47:139094..139138:−
21	scaffold22_27474	0	16 +/− 15%	2	Ucgggcgagacgagugcuuucc	scaffold22:336752..336810:+
22	scaffold1_2114	0	16 +/− 15%	3	Ccugucgcugcucgugaagccucu	scaffold1:2908975..2909036:+

**Table 2:** Gene targets of *Rhizoctonia solani*-milRNAs. These target genes encode carbohydrate active enzymes and secretory proteins of fungus.

milRNA Id	Target Gene	Gene Description
Carbohydrate Active Enzymes		
Rhi-milR-56	AG1IA_01218	Beta-glucosidase (EC 3.2.1.21)
Rhi-milR-120	AG1IA_01405	1,3-beta-glucan synthase component GLS2
Rhi-milR-122	AG1IA_01406	Sterol 3-beta-glucosyltransferase (EC 2.4.1.173) (Autophagy-related protein 26)
Rhi-milR-91	AG1IA_02027	Endoplasmic reticulum protein
Rhi-milR-169	AG1IA_02441	Beta-xylosidase
Rhi-milR-146	AG1IA_02513	Chitin deacetylase
Rhi-milR-120	AG1IA_02835	Putative 1,4-alpha-glucan branching enzyme from glycoside hydrolase family GH13
Rhi-milR-141	AG1IA_02889	Pectinesterase (EC 3.1.1.11)
Rhi-milR-150	AG1IA_03463	Glycosyltransferase family 2 protein
Rhi-milR-36	AG1IA_03939	Beta-mannosidase
Rhi-milR-91	AG1IA_04214	Trehalose 6-phosphate phosphatase, glycosyltransferase family 20 protein
Rhi-milR-81	AG1IA_04527	Uridine Di Phosphate-*N*-acetylglucosaminyltransferase
Rhi-milR-68	AG1IA_04727	Farnesyltransferase subunit beta
Rhi-milR-111	AG1IA_04740	Polysaccharide lyase family 1 protein
Rhi-milR-27	AG1IA_04862	1,3-beta-glucan synthase component GLS2
Rhi-milR-45	AG1IA_05653	Glycoside hydrolase family 51 protein
Rhi-milR-52	AG1IA_05719	Chitin synthase D
Rhi-milR-119	AG1IA_05754	Glycogen phosphorylase
Rhi-milR-42	AG1IA_05803	Alpha-galactosidase (EC 3.2.1.22) (Melibiase)
Rhi-milR-139	AG1IA_05807	Adenylosuccinate synthetase (AMPSase) (AdSS) (EC 6.3.4.4) (IMP-aspartate ligase)
Rhi-milR-122	AG1IA_06014	Glycoside hydrolase family 51 protein
Rhi-milR-41	AG1IA_06294	Glycoside hydrolase family 3 protein
Rhi-milR-58	AG1IA_06593	Alpha glucosidase II, alpha subunit, putative
Rhi-milR-167	AG1IA_07255	Killer toxin alpha/beta
Rhi-milR-51	AG1IA_07341	Galactan 1,3-beta-galactosidase
Rhi-milR-120	AG1IA_07743	Pectinesterase (EC 3.1.1.11)
Rhi-milR-162	AG1IA_07787	Glycoside hydrolase family 31 protein
Rhi-milR-168	AG1IA_07905	Glycoside hydrolase family 95 protein
Rhi-milR-97	AG1IA_08771	Exo-beta-1,3-glucanase
Secretory Proteins		
Rhi-milR-150	AG1IA_00157	Polysaccharide deacetylase domain-containing protein
Rhi-milR-98	AG1IA_01858	Uncharacterized protein
Rhi-milR-124	AG1IA_01958	Rad1 domain-containing protein
Rhi-milR-95	AG1IA_02532	Lipase domain-containing protein
Rhi-milR-144	AG1IA_03100	Glycosyl hydrolase family 61 domain-containing protein
Rhi-milR-122	AG1IA_03118	Uncharacterized protein
Rhi-milR-141	AG1IA_03171	Copper/zinc superoxide dismutase domain-containing protein
Rhi-milR-165	AG1IA_05741	Uncharacterized protein
Rhi-milR-141	AG1IA_06494	Uncharacterized protein
Rhi-milR-122	AG1IA_07117	Uncharacterized protein
Rhi-milR-52	AG1IA_07216	Uncharacterized protein
Rhi-milR-19	AG1IA_07698	Transcription initiation factor TFIID complex 60 kDa subunit
Rhi-milR-54	AG1IA_08056	Tyrosinase domain-containing protein
Rhi-milR-144	AG1IA_08227	Uncharacterized protein
Rhi-milR-90	AG1IA_08293	Cytochrome P450 domain-containing protein
Rhi-milR-146	AG1IA_08653	Uncharacterized protein
Rhi-milR-122	AG1IA_08711	Uncharacterized protein
Rhi-milR-144	AG1IA_09802	Protein tyrosine kinase domain-containing protein
Rhi-milR-130	AG1IA_10060	Cytochrome P450 domain-containing protein

**Table 3:** Target genes of *Rhizoctonia solani* novel milRNAs.

S. No.	Novel milRNA	Target Gene	Gene Description
1	scaffold47_37407	AG1IA_04067	Ubiquitin conjugating enzyme family protein
2	scaffold47_37407	AG1IA_02707	MFS transporter, putative
3	scaffold47_37523	AG1IA_04067	Ubiquitin conjugating enzyme family protein
4	scaffold47_37523	AG1IA_02707	MFS transporter, putative
5	scaffold47_37341	AG1IA_04067	Ubiquitin conjugating enzyme family protein
6	scaffold47_37341	AG1IA_02707	MFS transporter, putative
7	scaffold47_36931	AG1IA_08169	Phosphatidylinositol 3-kinase tor2
8	scaffold47_36931	AG1IA_05744	DNA-directed RNA polymerase subunit (EC 2.7.7.6)
9	scaffold47_36931	AG1IA_06882	EOS1 domain-containing protein
10	scaffold47_36931	AG1IA_06165	ABC transporter
11	scaffold47_36931	AG1IA_07629	Uncharacterized protein
12	scaffold47_36931	AG1IA_07663	ATP-dependent rRNA helicase RRP3
13	scaffold47_36931	AG1IA_04769	Fungal zn(2)-Cys(6) binuclear cluster domain-containing protein
14	scaffold47_36931	AG1IA_08613	BMR1 protein
15	scaffold47_36931	AG1IA_00053	Uncharacterized protein
16	scaffold47_36931	AG1IA_06822	Uncharacterized protein
17	scaffold47_36931	AG1IA_07843	Uncharacterized protein
18	scaffold47_36931	AG1IA_08221	Molybdenum cofactor biosynthesis protein
19	scaffold145_44745	AG1IA_06624	Uncharacterized protein
20	scaffold145_44745	AG1IA_10187	RNase H domain-containing protein
21	scaffold145_44745	AG1IA_10310	TFIIA domain-containing protein
22	scaffold145_44745	AG1IA_02688	GPI transamidase component PIG-S
23	scaffold145_44745	AG1IA_05493	Uncharacterized protein
24	scaffold145_44745	AG1IA_04447	HLH domain-containing protein

Abbreviations: MFS—Major Facilitator Superfamily, TFIIA—Transcription Factor IIA, GPI—Glycosylphosphatidylinositol, PIG-S—Phosphatidylinositol glycan biosynthesis class S protein, HLH—Helix Loop Helix.

**Table 4:** Target genes of *Rhizoctonia solani* milRNAs in rice genome; 24 milRNAs showed their target as rice genes encoding proteins associated with defense and other metabolic processes.

milRNAs	Target Gene Accession	Expect	Gene Description	Inhibition
Rhi-milR-1	LOC_Os01g47740.2	3	cDNA|zinc finger, C3HC4 type domain containing protein, expressed	Translation
Rhi-milR-111	LOC_Os07g48720.3	2.5	cDNA|MAG2, putative, expressed	Cleavage
Rhi-milR-124	LOC_Os09g23200.1	2.5	cDNA|KANADI1, putative, expressed	Cleavage
Rhi-milR-13	LOC_Os10g20630.1	1	cDNA|vacuolar-sorting receptor precursor, putative, expressed	Cleavage
	LOC_Os11g02464.1	2.5	cDNA|vacuolar-sorting receptor precursor, putative, expressed	Cleavage
Rhi-milR-130	LOC_Os06g16140.1	2.5	cDNA|expressed protein	Cleavage
Rhi-milR-131	LOC_Os12g42400.4	2.5	cDNA|nuclear transcription factor Y subunit, putative, expressed	Cleavage
	LOC_Os12g42400.1	2.5	cDNA|nuclear transcription factor Y subunit, putative, expressed	Cleavage
	LOC_Os02g58790.5	3	cDNA|cell division inhibitor, putative, expressed	Cleavage
	LOC_Os02g58790.2	3	cDNA|cell division inhibitor, putative, expressed	Cleavage
	LOC_Os02g58790.1	3	cDNA|cell division inhibitor, putative, expressed	Cleavage
	LOC_Os02g58790.4	3	cDNA|cell division inhibitor, putative, expressed	Cleavage
	LOC_Os02g58790.3	3	cDNA|cell division inhibitor, putative, expressed	Cleavage
	LOC_Os01g55200.1	3	cDNA|potassium channel KAT1, putative, expressed	Cleavage
Rhi-milR-135	LOC_Os12g16290.1	2.5	cDNA|isoflavone reductase, putative, expressed	Cleavage
	LOC_Os05g45180.1	3	cDNA|anthocyanidin 5,3-*O*-glucosyltransferase, putative, expressed	Cleavage
Rhi-milR-142	LOC_Os12g03740.1	2.5	cDNA|OsFBX438-F-box domain containing protein, expressed	Cleavage
Rhi-milR-159	LOC_Os12g43720.1	3	cDNA|early-responsive to dehydration protein-related, putative, expressed	Cleavage
Rhi-milR-160	LOC_Os03g24410.1	1.5	cDNA|expressed protein	Cleavage
Rhi-milR-18	LOC_Os08g30660.1	2.5	cDNA|NB-ARC domain containing protein, expressed	Cleavage
	LOC_Os12g02570.2	2.5	cDNA|expressed protein	Cleavage
	LOC_Os12g02570.1	2.5	cDNA|expressed protein	Cleavage
Rhi-milR-20	LOC_Os01g25430.1	2	cDNA|expressed protein	Cleavage
	LOC_Os07g48200.2	2.5	cDNA|B3 DNA binding domain containing protein, putative, expressed	Cleavage
	LOC_Os07g48200.1	2.5	cDNA|B3 DNA binding domain containing protein, putative, expressed	Cleavage
Rhi-milR-22	LOC_Os02g49350.1	2.5	cDNA|plastocyanin-like domain containing protein, putative, expressed	Cleavage
Rhi-milR-26	LOC_Os03g55250.1	2.5	cDNA|cytochrome P450 81E1, putative, expressed	Cleavage
Rhi-milR-43	LOC_Os04g28160.1	3	cDNA|response regulator receiver domain containing protein, expressed	Cleavage
Rhi-milR-51	LOC_Os04g34390.1	3	cDNA|serine/threonine-protein kinase receptor precursor, putative, expressed	Cleavage
Rhi-milR-61	LOC_Os10g26660.2	2.5	cDNA|expressed protein	Translation
	LOC_Os08g40620.1	2.5	cDNA|rabGAP/TBC domain-containing protein, putative, expressed	Cleavage
	LOC_Os08g40620.2	2.5	cDNA|rabGAP/TBC domain-containing protein, putative, expressed	Cleavage
Rhi-milR-66	LOC_Os05g11414.1	2.5	cDNA|OsMADS58-MADS-box family gene with MIKCc type-box, expressed	Cleavage
Rhi-milR-81	LOC_Os05g50910.2	3	cDNA|extra-large G-protein-related, putative, expressed	Cleavage
	LOC_Os05g50910.1	3	cDNA|extra-large G-protein-related, putative, expressed	Cleavage
Rhi-milR-89	LOC_Os06g33020.1	2.5	cDNA|retrotransposon protein, putative, Ty3-gypsy subclass, expressed	Cleavage
	LOC_Os06g32890.1	2.5	cDNA|retrotransposon protein, putative, Ty3-gypsy subclass, expressed	Cleavage
Rhi-milR-9	LOC_Os02g35820.2	2.5	cDNA|retrotransposon protein, putative, unclassified, expressed	Cleavage
	LOC_Os02g35820.1	2.5	cDNA|retrotransposon protein, putative, unclassified, expressed	Cleavage
	LOC_Os07g04230.1	2.5	cDNA|retrotransposon protein, putative, unclassified, expressed	Cleavage
Rhi-milR-97	LOC_Os07g47950.1	2.5	cDNA|expressed protein	Cleavage
scaffold130_44481	LOC_Os09g29390.1	2.5	cDNA|plastocyanin-like domain containing protein, putative, expressed	Cleavage
scaffold47_37559	LOC_Os04g52164.1	2	cDNA|transferase family protein, putative, expressed	Cleavage
	LOC_Os04g52164.2	2	cDNA|transferase family protein, putative, expressed	Cleavage

## Data Availability

All data generated or analysed during this study are included in this published article (and its Supplementary Information files). The sequence data is available on request.

## Supplementary Materials

The following are available online at https://www.mdpi.com/article/10.3390/jof7070561/s1, Table S1: List of primers used in this study for qRT-PCR assays. Table S2: List of known milRNAs identified in this study. Table S3: List of target genes of known and novel milRNAs. Table S4: Gene ontology analysis of selected target genes.
